# Rubidium 2,4,6-trioxo-1,3-diazinan-5-ide–1,3-diazinane-2,4,6-trione–water (1/1/1)

**DOI:** 10.1107/S1600536811012657

**Published:** 2011-04-13

**Authors:** Marlena Gryl, Katarzyna Stadnicka

**Affiliations:** a Faculty of Chemistry, Jagiellonian University, Ingardena 3, 30-060 Kraków, Poland

## Abstract

The asymmetric unit of the title compound, Rb^+^·C_4_H_3_N_2_O_3_^−^·C_4_H_4_N_2_O_3_·H_2_O, consists of one rubidium cation, a barbituric acid mol­ecule, a barbiturate anion and one water mol­ecule. The rubidium ion has seven close-contact inter­actions with O atoms, with Rb⋯O distances ranging from 2.8594 (16) to 3.2641 (14) Å. These seven O atoms together with an eighth O atom at 3.492 (2) Å away from Rb form a distorted polyhedron with shape inter­mediate between an anti­prism and a dodeca­hedron. The Rb^+^ ions connect layers built of organic components and water mol­ecules linked *via* N—H⋯O and O—H⋯O hydrogen bonds.

## Related literature

For the crystal structures of selected barbiturates, see: Xiong *et al.* (2003 ▶); Gryl *et al.* (2008 ▶, 2011 ▶); Braga *et al.* (2010 ▶); Garcia *et al.* (2010 ▶); Ivanova & Spiteller (2010 ▶) and for those of rubidium salts, see: Clegg & Liddle (2004 ▶); Yıldırım *et al.* (2008 ▶). For classification of hydrogen-bond systems according to graph-set theory, see: Bernstein *et al.* (1995 ▶).
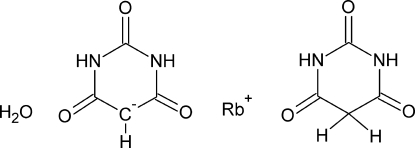


## Experimental

### 

#### Crystal data

Rb^+^·C_4_H_3_N_2_O_3_^−^·C_4_H_4_N_2_O_3_·H_2_O
*M* *_r_* = 358.66
Monoclinic, 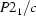

*a* = 9.8810 (1) Å
*b* = 19.6790 (5) Å
*c* = 6.4530 (3) Å
β = 108.26 (2)°
*V* = 1191.59 (15) Å^3^
*Z* = 4
Mo *K*α radiation
μ = 4.20 mm^−1^
*T* = 293 K
0.43 × 0.23 × 0.21 mm

#### Data collection

Nonius KappaCCD diffractometer
Absorption correction: multi-scan (*DENZO* and *SCALEPACK*; Otwinowski & Minor, 1997 ▶) *T*_min_ = 0.266, *T*_max_ = 0.473
17623 measured reflections
2555 independent reflections
2239 reflections with *I* > 2σ(*I*)
*R*_int_ = 0.037

#### Refinement

*R*[*F* ^2^ > 2σ(*F* ^2^)] = 0.024
*wR*(*F* ^2^) = 0.059
*S* = 1.03
2555 reflections
199 parameters
6 restraints
H atoms treated by a mixture of independent and constrained refinement
Δρ_max_ = 0.27 e Å^−3^
Δρ_min_ = −0.30 e Å^−3^

### 

Data collection: *COLLECT* (Nonius, 1998 ▶); cell refinement: *SCALEPACK* (Otwinowski & Minor, 1997 ▶); data reduction: *DENZO* (Otwinowski & Minor, 1997 ▶) and *SCALEPACK*; program(s) used to solve structure: *SIR92* (Altomare *et al.*, 1994 ▶); program(s) used to refine structure: *SHELXL97* (Sheldrick, 2008 ▶); molecular graphics: *Mercury* (Macrae *et al.*, 2006 ▶) and *ORTEP-3* (Farrugia, 1997 ▶); software used to prepare material for publication: *SHELXL97* and *WinGX* (Farrugia, 1999 ▶).

## Supplementary Material

Crystal structure: contains datablocks global, I. DOI: 10.1107/S1600536811012657/vm2087sup1.cif

Structure factors: contains datablocks I. DOI: 10.1107/S1600536811012657/vm2087Isup2.hkl

Additional supplementary materials:  crystallographic information; 3D view; checkCIF report

## Figures and Tables

**Table 1:** Hydrogen-bond geometry (Å, °)

*D*—H⋯*A*	*D*—H	H⋯*A*	*D*⋯*A*	*D*—H⋯*A*
N1*A*—H1*A*⋯O6*B*^i^	0.88 (1)	1.90 (1)	2.769 (2)	172 (2)
N3*A*—H3*A*⋯O4*B*	0.86 (1)	1.84 (1)	2.694 (2)	175 (2)
N1*B*—H1*B*⋯O2*A*^ii^	0.88 (1)	1.94 (1)	2.820 (2)	175 (2)
N3*B*—H3*B*⋯O4*A*	0.87 (1)	2.12 (1)	2.975 (2)	169 (2)
O1*W*—H1*W*⋯O6*B*^iii^	0.84 (1)	1.87 (1)	2.700 (2)	171 (2)
O1*W*—H2*W*⋯O4*A*^iv^	0.83 (1)	2.08 (1)	2.898 (2)	170 (3)

Symmetry codes: (i) 
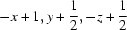
; (ii) 
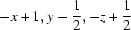
; (iii) 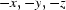; (iv) 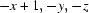.

**Fig. 1.**
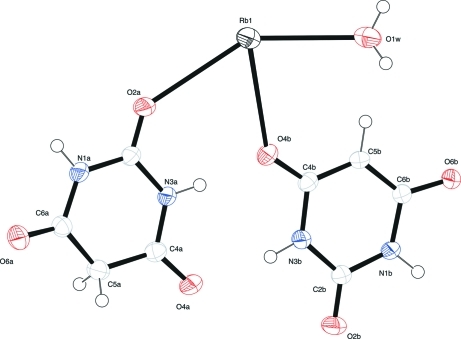
Asymmetric unit of the title addition compound showing displacement ellipsoids drawn at the 50% probability level (H atoms are shown as spheres of arbitrary radii). The atoms of barbituric acid molecule are marked by the letter a, whereas those of barbiturate anion with the letter b.

**Fig. 2.**
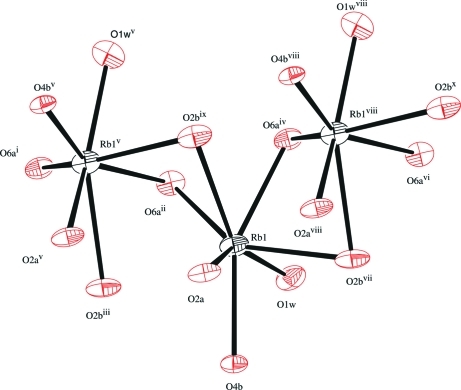
Rubidium polyhedra of Rb1v, Rb1 and Rb1viii joined by edges O6aii, O2bix and O2bvii, O6aiv with Rb—Rb distance of 4.1988 (3) Å. Symmetry codes: (i) x - 1, -y + 1/2, z + 1/2; (ii) x - 1, y, z; (iii) -x + 1, -y, -z + 1; (iv) x - 1, -y + 1/2, z - 1/2; (v) x, -y + 1/2, z + 1/2; (vi) x - 1, y, z - 1; (vii) -x + 1, -y, -z; (viii) x, -y + 1/2, z - 1/2; (ix) -x + 1, y + 1/2, -z + 1/2; (x) -x + 1, y + 1/2, -z - 1/2.

**Fig. 3.**
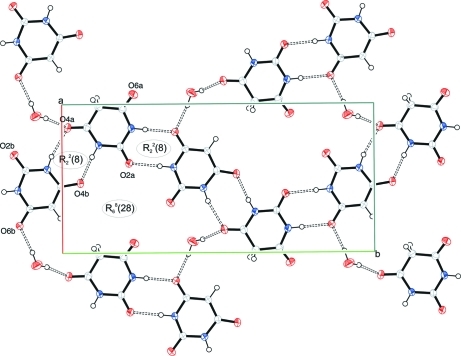
Hydrogen bond scheme in the organic layer parallel to ab at z = 0.25. Hydrogen bond graph-set descriptors R22(8) (two kinds) and R86(28) are given according to Bernstein et al., (1995).

**Fig. 4.**
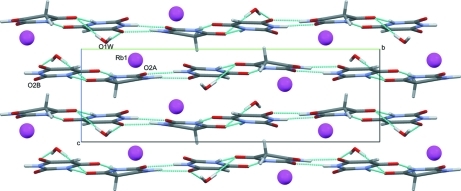
View of the packing along [100] showing the Rb cations in between the layers of organic components and water molecules.

## supplementary crystallographic information

### Comment

Recently we have reported structures for three polymorphic forms of barbituric acid and urea addition compounds (Gryl *et al.*, 2008) for two of which a charge density analysis was also performed (Gryl *et al.*, 2011). Barbituric acid appeared as a valuable component in designing new, functional materials and in particular polar materials (Xiong, *et al.*, 2003). However many attempts to design and obtain polar barbiturates failed (see for example Ivanova & Spiteller, 2010). Herein we report the structure of the title addition compound (I), the asymmetric unit of which is comprised of a rubidium cation, barbiturate anion, barbituric acid molecule and one water molecule (Fig. 1). Unfortunately, like for many barbiturates, the structure is centrosymmetric (space group *P*2_1_/*c*). Each Rb1 cation is surrounded by seven oxygen atoms and bridged by O6a (*x* - 1, *y*, *z*) and O2b (-*x* + 1, *y* + 1/2, -*z* + 1/2) to Rb1 (*x*, -*y+1/2*, *z* + 1/2) and by O2b (*x*, -*y* + 1/2, *z* - 1/2) and O6a (*x-*1, -*y* + 1/2, *z* - 1/2) to Rb1 (*x*, -*y* + 1/2, *z* - 1/2) (Fig. 2).

All barbiturate NH groups and water molecules act as hydrogen bond donors. The hydrogen bond geometry is given in Table 1. There are considerable differences in the accepting properties of the carbonyl oxygen atoms. In the barbituric acid molecule, only atom O4a is a hydrogen bond acceptor from O1W, whereas atoms O2a and O6a interact with Rb ions. A different situation is observed in the barbituriate ion: atom O6b is an acceptor of two hydrogen bonds from O1W and N1a whereas atoms O2b and O4b are both involved in interactions with rubidium ions. The structure is comprised of layers built of barbituric acid molecules and barbiturate anions connected by hydrogen bonds (Fig. 3). Graph-set descriptors *R*_2_^2^(8) and *R*_8_^6^(28) were assigned to the hydrogen bonds according to Bernstein *et al.*, (1995). The two ring systems of *R*_2_^2^(8) are formed between barbiturate anions and barbituric acid molecules by crystallographically different hydrogen bonds. In the *R*_8_^6^(28) ring formation additionally two water molecules act as hydrogen bond donors. The layers, parallel to *ab*, are joined together into a three dimensional structure due to interactions of Rb1 cations with oxygen atoms from barbiturate anions, barbituric acid molecules and water molecules (Fig. 4).

### Experimental

The title compound was synthesized by mixing aqueous solutions of barbituric acid and rubidium carbonate prepared at 323 K using a water bath. Single crystals suitable for X-ray diffraction were obtained from ethanol solution by slow evaporation at ambient conditions.

### Refinement

All hydrogen atoms of N—H and O—H groups were found in difference Fourier maps and refined in a riding model assuming N—H = 0.88 (1) Å, O—H = 0.84 (1) Å and *U*_iso_ = 1.2*U*_eq_ of the parent atom. Hydrogen atoms of CH and CH_2_ groups were found in difference Fourier maps and refined from geometrical positions assuming C—H = 0.97 Å for CH and C—H = 0.93 Å for CH_2_ groups and using riding model with *U*_iso_=1.2*U*_eq_ (C5A and C5B, respectively).

### Crystal data

**Table:**

Rb^+^·C_4_H_4_N_2_O_3_^−^·C_4_H_5_N_2_O_3_·H_2_O	*F*(000) = 712
*M**_r_* = 358.66	*D*_x_ = 1.999 Mg m^−^^3^
Monoclinic, *P*2_1_/*c*	Mo *K*α radiation, λ = 0.71073 Å
Hall symbol: -P 2ybc	Cell parameters from 3522 reflections
*a* = 9.8810 (1) Å	θ = 1.0–30.0°
*b* = 19.6790 (5) Å	µ = 4.20 mm^−^^1^
*c* = 6.4530 (3) Å	*T* = 293 K
β = 108.26 (2)°	Block, colorless
*V* = 1191.59 (15) Å^3^	0.43 × 0.23 × 0.21 mm
*Z* = 4	

### Data collection

**Table:**

Nonius KappaCCD diffractometer	2555 independent reflections
Radiation source: fine-focus sealed tube	2239 reflections with *I* > 2σ(*I*)
horizontally mounted graphite crystal	*R*_int_ = 0.037
Detector resolution: 9 pixels mm^-1^	θ_max_ = 27.0°, θ_min_ = 3.5°
φ and ω scans to fill Ewald sphere	*h* = −12→11
Absorption correction: multi-scan (*DENZO* and *SCALEPACK*; Otwinowski & Minor, 1997)	*k* = 0→25
*T*_min_ = 0.266, *T*_max_ = 0.473	*l* = 0→8
17623 measured reflections	

### Refinement

**Table:**

Refinement on *F*^2^	Secondary atom site location: difference Fourier map
Least-squares matrix: full	Hydrogen site location: difference Fourier map
*R*[*F*^2^ > 2σ(*F*^2^)] = 0.024	H atoms treated by a mixture of independent and constrained refinement
*wR*(*F*^2^) = 0.059	*w* = 1/[σ^2^(*F*_o_^2^) + (0.0308*P*)^2^ + 0.3427*P*] where *P* = (*F*_o_^2^ + 2*F*_c_^2^)/3
*S* = 1.03	(Δ/σ)_max_ < 0.001
2555 reflections	Δρ_max_ = 0.27 e Å^−^^3^
199 parameters	Δρ_min_ = −0.30 e Å^−^^3^
6 restraints	Extinction correction: *SHELXL97* (Sheldrick, 2008)
0 constraints	Extinction coefficient: 0
Primary atom site location: difference Fourier map	

### Special details

**Table:**

Geometry. All e.s.d.'s (except the e.s.d. in the dihedral angle between two l.s. planes) are estimated using the full covariance matrix. The cell e.s.d.'s are taken into account individually in the estimation of e.s.d.'s in distances, angles and torsion angles; correlations between e.s.d.'s in cell parameters are only used when they are defined by crystal symmetry. An approximate (isotropic) treatment of cell e.s.d.'s is used for estimating e.s.d.'s involving l.s. planes.

### Fractional atomic coordinates and isotropic or equivalent isotropic displacement parameters (Å^2^)

**Table:**

	*x*	*y*	*z*	*U*_iso_*/*U*_eq_	
Rb1	0.291837 (19)	0.181732 (10)	0.12045 (3)	0.03861 (8)	
N1A	0.83901 (16)	0.22220 (7)	0.3062 (2)	0.0284 (3)	
H1A	0.830 (2)	0.2666 (5)	0.295 (3)	0.034*	
C2A	0.71713 (19)	0.18635 (8)	0.2905 (3)	0.0262 (4)	
O2A	0.60363 (14)	0.21479 (6)	0.2672 (2)	0.0390 (3)	
N3A	0.72641 (16)	0.11731 (7)	0.2984 (2)	0.0267 (3)	
H3A	0.6475 (14)	0.0959 (9)	0.281 (3)	0.032*	
C4A	0.84786 (19)	0.08055 (9)	0.3319 (3)	0.0269 (4)	
O4A	0.84464 (14)	0.01867 (6)	0.3376 (2)	0.0368 (3)	
C5A	0.98315 (19)	0.11930 (9)	0.3628 (3)	0.0310 (4)	
H5A1	1.0442	0.1123	0.5115	0.037*	
H5A2	1.0314	0.0999	0.2670	0.037*	
C6A	0.96859 (19)	0.19423 (9)	0.3212 (3)	0.0272 (4)	
O6A	1.06609 (14)	0.22942 (7)	0.3061 (2)	0.0375 (3)	
N1B	0.42573 (15)	−0.14265 (7)	0.2580 (2)	0.0289 (3)	
H1B	0.417 (2)	−0.1870 (5)	0.259 (3)	0.035*	
C2B	0.55857 (18)	−0.11709 (8)	0.2913 (3)	0.0280 (4)	
O2B	0.66394 (15)	−0.15406 (7)	0.3276 (3)	0.0447 (4)	
N3B	0.56512 (15)	−0.04808 (7)	0.2804 (2)	0.0267 (3)	
H3B	0.6520 (12)	−0.0339 (10)	0.304 (3)	0.032*	
C4B	0.44849 (18)	−0.00466 (8)	0.2415 (3)	0.0246 (3)	
O4B	0.47093 (14)	0.05801 (6)	0.2357 (2)	0.0325 (3)	
C5B	0.31602 (18)	−0.03460 (8)	0.2126 (3)	0.0266 (4)	
H5B	0.2358	−0.0073	0.1893	0.032*	
C6B	0.30235 (18)	−0.10459 (9)	0.2180 (3)	0.0256 (3)	
O6B	0.18751 (13)	−0.13782 (6)	0.1870 (2)	0.0378 (3)	
O1W	0.07639 (16)	0.08325 (8)	−0.0724 (3)	0.0478 (4)	
H1W	−0.0078 (14)	0.0973 (12)	−0.120 (4)	0.057*	
H2W	0.090 (3)	0.0514 (9)	−0.147 (4)	0.057*	

### Atomic displacement parameters (Å^2^)

**Table:**

	*U*^11^	*U*^22^	*U*^33^	*U*^12^	*U*^13^	*U*^23^
Rb1	0.02804 (11)	0.03333 (12)	0.05520 (14)	0.00282 (7)	0.01410 (9)	0.00032 (8)
N1A	0.0278 (8)	0.0187 (7)	0.0413 (8)	−0.0023 (6)	0.0148 (6)	−0.0005 (6)
C2A	0.0261 (9)	0.0205 (8)	0.0338 (9)	−0.0004 (6)	0.0120 (7)	−0.0006 (7)
O2A	0.0269 (7)	0.0209 (6)	0.0718 (9)	0.0017 (5)	0.0194 (6)	0.0009 (6)
N3A	0.0232 (7)	0.0176 (7)	0.0412 (8)	−0.0019 (6)	0.0127 (6)	−0.0002 (6)
C4A	0.0291 (9)	0.0251 (8)	0.0274 (8)	0.0020 (7)	0.0102 (7)	0.0018 (7)
O4A	0.0335 (7)	0.0198 (6)	0.0569 (8)	0.0035 (5)	0.0138 (6)	0.0021 (6)
C5A	0.0260 (9)	0.0284 (9)	0.0394 (9)	0.0028 (7)	0.0115 (7)	0.0047 (7)
C6A	0.0253 (9)	0.0282 (9)	0.0289 (8)	−0.0017 (7)	0.0098 (7)	−0.0009 (7)
O6A	0.0288 (7)	0.0344 (7)	0.0528 (8)	−0.0062 (6)	0.0177 (6)	0.0006 (6)
N1B	0.0239 (7)	0.0164 (7)	0.0452 (8)	0.0016 (6)	0.0093 (6)	0.0015 (6)
C2B	0.0240 (9)	0.0223 (8)	0.0376 (9)	0.0012 (7)	0.0095 (7)	0.0010 (7)
O2B	0.0271 (7)	0.0270 (7)	0.0791 (10)	0.0074 (6)	0.0152 (7)	0.0045 (7)
N3B	0.0218 (7)	0.0217 (7)	0.0373 (8)	−0.0005 (6)	0.0102 (6)	0.0007 (6)
C4B	0.0276 (9)	0.0208 (8)	0.0245 (8)	0.0013 (6)	0.0071 (6)	0.0010 (6)
O4B	0.0317 (7)	0.0185 (6)	0.0468 (7)	−0.0007 (5)	0.0115 (6)	0.0031 (5)
C5B	0.0232 (8)	0.0205 (8)	0.0342 (9)	0.0042 (6)	0.0061 (7)	0.0004 (7)
C6B	0.0229 (8)	0.0223 (8)	0.0293 (8)	0.0005 (6)	0.0050 (6)	−0.0011 (7)
O6B	0.0221 (6)	0.0215 (6)	0.0661 (9)	−0.0010 (5)	0.0085 (6)	−0.0024 (6)
O1W	0.0315 (8)	0.0473 (9)	0.0610 (10)	0.0102 (7)	0.0094 (7)	−0.0118 (7)

### Geometric parameters (Å, °)

**Table:**

Rb1—O1W	2.8594 (16)	O6A—Rb1^v^	2.9942 (13)
Rb1—O4B	2.9645 (12)	O6A—Rb1^vi^	3.0517 (13)
Rb1—O6A^i^	2.9942 (13)	N1B—C2B	1.358 (2)
Rb1—O2A	2.9972 (13)	N1B—C6B	1.384 (2)
Rb1—O6A^ii^	3.0517 (13)	N1B—H1B	0.878 (9)
Rb1—O2B^iii^	3.1049 (16)	C2B—O2B	1.231 (2)
Rb1—O2B^iv^	3.2641 (14)	C2B—N3B	1.363 (2)
N1A—C6A	1.369 (2)	O2B—Rb1^iii^	3.1049 (16)
N1A—C2A	1.372 (2)	O2B—Rb1^vii^	3.2641 (14)
N1A—H1A	0.879 (9)	O2B—Rb1^viii^	3.4923 (16)
C2A—O2A	1.220 (2)	N3B—C4B	1.393 (2)
C2A—N3A	1.362 (2)	N3B—H3B	0.870 (9)
N3A—C4A	1.359 (2)	C4B—O4B	1.256 (2)
N3A—H3A	0.862 (9)	C4B—C5B	1.394 (2)
C4A—O4A	1.219 (2)	C5B—C6B	1.386 (2)
C4A—C5A	1.497 (2)	C5B—H5B	0.9300
C5A—C6A	1.498 (2)	C6B—O6B	1.270 (2)
C5A—H5A1	0.9700	O1W—H1W	0.839 (10)
C5A—H5A2	0.9700	O1W—H2W	0.828 (10)
C6A—O6A	1.215 (2)		
			
O1W—Rb1—O4B	81.78 (4)	C6A—N1A—H1A	118.1 (14)
O1W—Rb1—O6A^i^	80.80 (4)	C2A—N1A—H1A	116.4 (14)
O4B—Rb1—O6A^i^	128.17 (4)	O2A—C2A—N3A	120.73 (16)
O1W—Rb1—O2A	146.72 (4)	O2A—C2A—N1A	121.67 (15)
O4B—Rb1—O2A	68.00 (3)	N3A—C2A—N1A	117.59 (15)
O6A^i^—Rb1—O2A	128.79 (4)	C2A—O2A—Rb1	138.91 (11)
O1W—Rb1—O6A^ii^	79.02 (4)	C4A—N3A—C2A	125.64 (15)
O4B—Rb1—O6A^ii^	153.12 (4)	C4A—N3A—H3A	118.6 (14)
O6A^i^—Rb1—O6A^ii^	66.76 (3)	C2A—N3A—H3A	115.8 (14)
O2A—Rb1—O6A^ii^	123.40 (4)	O4A—C4A—N3A	120.43 (16)
O1W—Rb1—O2B^iii^	77.21 (4)	O4A—C4A—C5A	122.37 (16)
O4B—Rb1—O2B^iii^	80.94 (4)	N3A—C4A—C5A	117.20 (15)
O6A^i^—Rb1—O2B^iii^	140.12 (4)	C6A—C5A—C4A	116.49 (15)
O2A—Rb1—O2B^iii^	84.36 (4)	C6A—C5A—H5A1	108.2
O6A^ii^—Rb1—O2B^iii^	76.60 (4)	C4A—C5A—H5A1	108.2
O1W—Rb1—O2B^iv^	140.64 (4)	C6A—C5A—H5A2	108.2
O4B—Rb1—O2B^iv^	137.49 (4)	C4A—C5A—H5A2	108.2
O6A^i^—Rb1—O2B^iv^	75.00 (4)	H5A1—C5A—H5A2	107.3
O2A—Rb1—O2B^iv^	70.21 (3)	O6A—C6A—N1A	120.85 (16)
O6A^ii^—Rb1—O2B^iv^	63.09 (4)	O6A—C6A—C5A	122.79 (16)
O2B^iii^—Rb1—O2B^iv^	102.47 (4)	N1A—C6A—C5A	116.33 (15)
O1W—Rb1—O2B^viii^	100.15 (4)	C6A—O6A—Rb1^v^	124.21 (12)
O4B—Rb1—O2B^viii^	75.04 (3)	C6A—O6A—Rb1^vi^	134.92 (12)
O6A^i^—Rb1—O2B^viii^	60.78 (3)	Rb1^v^—O6A—Rb1^vi^	87.97 (3)
O2A—Rb1—O2B^viii^	85.63 (4)	C2B—N1B—C6B	125.45 (15)
O6A^ii^—Rb1—O2B^viii^	126.81 (3)	C2B—N1B—H1B	117.6 (15)
O2B^iii^—Rb1—O2B^viii^	155.95 (5)	C6B—N1B—H1B	116.9 (15)
O2B^iv^—Rb1—O2B^viii^	94.58 (4)	O2B—C2B—N1B	121.94 (16)
O1W—Rb1—C2B^iii^	82.13 (4)	O2B—C2B—N3B	123.22 (16)
O4B—Rb1—C2B^iii^	63.29 (4)	N1B—C2B—N3B	114.84 (15)
O6A^i^—Rb1—C2B^iii^	157.33 (4)	O2B—C2B—Rb1^iii^	54.65 (10)
O2A—Rb1—C2B^iii^	72.24 (4)	N1B—C2B—Rb1^iii^	110.33 (11)
O6A^ii^—Rb1—C2B^iii^	95.40 (4)	N3B—C2B—Rb1^iii^	105.84 (11)
O2B^iii^—Rb1—C2B^iii^	18.87 (4)	C2B—O2B—Rb1^iii^	106.49 (12)
O2B^iv^—Rb1—C2B^iii^	110.36 (4)	C2B—O2B—Rb1^vii^	133.05 (12)
O2B^viii^—Rb1—C2B^iii^	137.58 (4)	Rb1^iii^—O2B—Rb1^vii^	82.45 (3)
O1W—Rb1—Rb1^ix^	126.08 (3)	C2B—O2B—Rb1^viii^	96.57 (11)
O4B—Rb1—Rb1^ix^	118.45 (2)	Rb1^iii^—O2B—Rb1^viii^	155.95 (5)
O6A^i^—Rb1—Rb1^ix^	46.58 (3)	Rb1^vii^—O2B—Rb1^viii^	76.76 (3)
O2A—Rb1—Rb1^ix^	82.29 (3)	C2B—N3B—C4B	124.81 (15)
O6A^ii^—Rb1—Rb1^ix^	88.18 (3)	C2B—N3B—H3B	111.8 (14)
O2B^iii^—Rb1—Rb1^ix^	149.52 (3)	C4B—N3B—H3B	123.4 (14)
O2B^iv^—Rb1—Rb1^ix^	47.14 (3)	O4B—C4B—N3B	117.65 (15)
O2B^viii^—Rb1—Rb1^ix^	49.18 (2)	O4B—C4B—C5B	125.34 (16)
C2B^iii^—Rb1—Rb1^ix^	151.63 (3)	N3B—C4B—C5B	117.01 (15)
O1W—Rb1—Rb1^x^	106.17 (3)	C4B—O4B—Rb1	135.81 (11)
O4B—Rb1—Rb1^x^	125.08 (2)	C6B—C5B—C4B	120.79 (15)
O6A^i^—Rb1—Rb1^x^	106.63 (3)	C6B—C5B—H5B	119.6
O2A—Rb1—Rb1^x^	81.74 (3)	C4B—C5B—H5B	119.6
O6A^ii^—Rb1—Rb1^x^	45.45 (3)	O6B—C6B—C5B	126.67 (16)
O2B^iii^—Rb1—Rb1^x^	50.41 (3)	O6B—C6B—N1B	116.23 (15)
O2B^iv^—Rb1—Rb1^x^	54.06 (3)	C5B—C6B—N1B	117.09 (15)
O2B^viii^—Rb1—Rb1^x^	148.58 (2)	Rb1—O1W—H1W	117.0 (18)
C2B^iii^—Rb1—Rb1^x^	64.29 (3)	Rb1—O1W—H2W	122.1 (19)
Rb1^ix^—Rb1—Rb1^x^	100.428 (8)	H1W—O1W—H2W	111 (3)
C6A—N1A—C2A	125.34 (15)		
			
C6A—N1A—C2A—O2A	−176.43 (17)	C6B—N1B—C2B—O2B	179.49 (17)
C6A—N1A—C2A—N3A	2.6 (3)	C6B—N1B—C2B—N3B	−0.8 (3)
O2A—C2A—N3A—C4A	−177.35 (17)	O2B—C2B—N3B—C4B	−179.42 (17)
N1A—C2A—N3A—C4A	3.6 (3)	N1B—C2B—N3B—C4B	0.8 (2)
C2A—N3A—C4A—O4A	179.49 (17)	C2B—N3B—C4B—O4B	−179.93 (16)
C2A—N3A—C4A—C5A	0.0 (2)	C2B—N3B—C4B—C5B	0.2 (2)
O4A—C4A—C5A—C6A	171.78 (16)	O4B—C4B—C5B—C6B	178.77 (16)
N3A—C4A—C5A—C6A	−8.7 (2)	N3B—C4B—C5B—C6B	−1.4 (2)
C2A—N1A—C6A—O6A	170.35 (17)	C4B—C5B—C6B—O6B	−177.57 (17)
C2A—N1A—C6A—C5A	−11.3 (2)	C4B—C5B—C6B—N1B	1.4 (2)
C4A—C5A—C6A—O6A	−167.85 (17)	C2B—N1B—C6B—O6B	178.77 (17)
C4A—C5A—C6A—N1A	13.8 (2)	C2B—N1B—C6B—C5B	−0.3 (3)

Symmetry codes: (i) *x*−1, *y*, *z*; (ii) *x*−1, −*y*+1/2, *z*−1/2; (iii) −*x*+1, −*y*, −*z*; (iv) −*x*+1, *y*+1/2, −*z*+1/2; (v) *x*+1, *y*, *z*; (vi) *x*+1, −*y*+1/2, *z*+1/2; (vii) −*x*+1, *y*−1/2, −*z*+1/2; (viii) −*x*+1, −*y*, −*z*+1; (ix) *x*, −*y*+1/2, *z*+1/2; (x) *x*, −*y*+1/2, *z*−1/2.

### Hydrogen-bond geometry (Å, °)

**Table:**

*D*—H···*A*	*D*—H	H···*A*	*D*···*A*	*D*—H···*A*
N1A—H1A···O6B^iv^	0.88 (1)	1.90 (1)	2.769 (2)	172 (2)
N3A—H3A···O4B	0.86 (1)	1.84 (1)	2.694 (2)	175 (2)
N1B—H1B···O2A^vii^	0.88 (1)	1.94 (1)	2.820 (2)	175 (2)
N3B—H3B···O4A	0.87 (1)	2.12 (1)	2.975 (2)	169 (2)
O1W—H1W···O6B^xi^	0.84 (1)	1.87 (1)	2.700 (2)	171 (2)
O1W—H2W···O4A^iii^	0.83 (1)	2.08 (1)	2.898 (2)	170 (3)

Symmetry codes: (iv) −*x*+1, *y*+1/2, −*z*+1/2; (vii) −*x*+1, *y*−1/2, −*z*+1/2; (xi) −*x*, −*y*, −*z*; (iii) −*x*+1, −*y*, −*z*.
